# Optimal Robust Control of Nonlinear Systems with Unknown Dynamics via NN Learning with Relaxed Excitation

**DOI:** 10.3390/e26010072

**Published:** 2024-01-14

**Authors:** Rui Luo, Zhinan Peng, Jiangping Hu

**Affiliations:** 1 School of Automation Engineering, University of Electronic Science and Technology of China, Chengdu 611731, China; nicole9922@163.com (R.L.); hujp@uestc.edu.cn (J.H.); 2 Institute of Electronic and Information Engineering, University of Electronic Science and Technology of China, Dongguan 523808, China; 3 Yangtze Delta Region Institute (Huzhou), University of Electronic Science and Technology of China, Huzhou 313001, China

**Keywords:** optimal robust control, nonlinear systems, parameter estimation, neural networks learning, relaxed PE conditions

## Abstract

This paper presents an adaptive learning structure based on neural networks (NNs) to solve the optimal robust control problem for nonlinear continuous-time systems with unknown dynamics and disturbances. First, a system identifier is introduced to approximate the unknown system matrices and disturbances with the help of NNs and parameter estimation techniques. To obtain the optimal solution of the optimal robust control problem, a critic learning control structure is proposed to compute the approximate controller. Unlike existing identifier-critic NNs learning control methods, novel adaptive tuning laws based on Kreisselmeier’s regressor extension and mixing technique are designed to estimate the unknown parameters of the two NNs under relaxed persistence of excitation conditions. Furthermore, theoretical analysis is also given to prove the significant relaxation of the proposed convergence conditions. Finally, effectiveness of the proposed learning approach is demonstrated via a simulation study.

## 1. Introduction

In the past several decades, much attention has been given to $H_{\infty }$ control problems, wherein the aim is to eliminate the influence of disturbance on the system. $H_{\infty }$ control mainly focuses on designing a robust controller to regulate and stabilize the system. In practice, we should not only focus on the control performance, but also consider the optimization of the system [1,2]. Therefore, optimal $H_{\infty }$ control problems will always be a hot research topic.

Adaptive dynamic programming (ADP), as one of the optimal control methods, has emerged as a powerful tool through which to deal with the optimal control problems of all kinds of dynamic systems [3]. The ADP framework combines dynamic programming and neural network approximation, and it has strong learning and adaptive ability. In this sense, ADP has rapidly developed in the control community in recent years. Generally speaking, the core of controller designs mainly concentrates on solving a Hamilton–Jacobi–Bellman (HJB) equation for nonlinear systems or an algebraic Riccati equation for linear systems [4]. Unfortunately, the HJB equation contains nonlinear, partial differential parts, which are difficult to solve directly [5]. Therefore, many efforts have been made for finding approximate solutions to the HJB equation using iterative or learning methods. Regarding the case of iterative methods, the ADP can be classed into two categories: value iteration (VI) [6,7] and policy iteration (PI) [8,9]. Regarding the case of learning-based methods, neural network (NN) approximation is generally utilized to learn the optimal or suboptimal solutions to the HJB equation. The standard learning frameworks include the following: actor–critic NNs and only-critic NNs. However, the abovementioned pieces of literature require partial or full model information in the controller design loop. To avoid relying on system models, many data-driven or model-free methods have been developed for improving the existing ADP frameworks, that is, data-driven RL [7], integral RL (IRL) [10,11], and system identification-based ADP methods [12,13,14].

More recently, excellent development has been realized with the use of ADP for the robust controller designs of optimal $H_{\infty }$ control problems [15,16,17]. The main way through which to solve optimal $H_{\infty }$ control problems is to model such problems as a two-player zero-sum game (min–max optimization problem), where the controller and the disturbance are viewed as players that try to find a controller to minimize the performance index function in worst-case disturbance conditions [18,19]. However, the disadvantage of zero-sum games is in judging the existence of the saddle point, which is generally difficult to judged. In order to overcome this issue, an indirect method motivated by [20] was developed by formulating an optimal regulation for a nominal system with new designs of the cost/value function [21]. For instance, Yang et al. proposed an event-triggered robust control strategy for nonlinear systems [22] using the indirect method. Xue et al. studied a tracking control problem for partial continuous-time systems with uncertainties and constraints [23] by transforming the robust control problem into an optimal regulation of nominal systems.

However, the existing results on $H_{\infty }$ optimal control designs have two main characteristics: (1) their controller designs are based on the assumption that the complete or partial knowledge of the system dynamics are known in advance; however, (2) to address this issue, some system identification methods have been proposed, such as the identifier–critic- or identifier–actor–critic-based designs of $H_{\infty }$ optimal control. However, it is generally required that the persistence of excitation (PE) condition must be satisfied to ensure the learning performance of the weight updating of neural networks, which is difficult to check online in practice [18,19,23]. Therefore, how to weaken the PE condition is also the research motivation of this paper.

From the abovementioned observations and considerations, in this paper, we propose a novel online parameter estimation method based on an identifier–critic learning control framework for the $H_{\infty }$ optimal control of nonlinear systems that have unknown dynamics with relaxed PE conditions. The contributions of our work can be summarized as follows:

- A new online identifier–critic learning control framework with a relaxed PE condition is proposed to address robust control for unknown continuous-time systems subject to unknown disturbances. To reconstruct the information of the system dynamics, neural networks combined with the linear regressor method are established to approximate the unknown system dynamics and disturbances.
- The approach in this paper is different from the existing weight adaption laws [18,19,23], where the PE condition is needed to ensure the learning performance of the NN’s weight parameters. However, such a condition is difficult to check online, and a general way through which to satisfy this condition is to add external noise to the controller, which may lead to the instability of the system. To overcome this issue, a Kreisselmeier regressor extension and mixing (KREM)-based weight adaption law is designed for identifier–critic NNs with new convergence conditions.
- Weak PE properties of new convergence conditions are analyzed rigorously compared to traditional PE conditions. Moreover, the theoretical results indicate that the closed-loop system’s stability and the convergence of identifier–critic learning are guaranteed.

The remainder of this article is organized as follows. In Section 2, some preliminaries are introduced and the optimal robust control problem of nonlinear continuous-time systems is given. Then, a system identifier design with a relaxed PE condition is constructed in Section 3. Section 4 gives the critic NN design for robust control under a relaxed PE condition. Theoretical analyses of the weak PE properties under new convergence conditions and the stability of the closed-loop systems are given in Section 5. The simulation results are provided in Section 6. Some conclusions are summarized in Section 7.

## 2. Preliminaries and Problem Formulation

In this section, some notation and definitions are first introduced. Then, the optimal robust control problem of the nonlinear continuous-time systems is described.

### 2.1. Preliminaries

To facilitate readability, some notations are listed. 

λ(·)

Eigenvalue of a matrix

${\left\{\cdot \right\}}^{*}$

Adjoint matrix

$I_{n}$

Identity matrix

tr(·)

Trace of a matrix

$\lambda_{M}\left(\cdot \right)$

Maximum eigenvalues

$\lambda_{m}\left(\cdot \right)$

Minimum eigenvalues

The following definitions will be used in the sequel.

**Definition** **1** ( PersistenceofExcitation [24]). *A bounded signal ψ(t) is said to be PE, if there exist positive constants T and $\delta_{1}$ such that*

$$\int_{t}^{t+T}\psi \left(r\right)\psi^{T}\left(r\right)dr\geq \delta_{1}I.$$

*For clarity, we indicate that ψ(t) satisfies the PE condition using the notation ψ(t)∈PE ; otherwise, ψ(t)∉PE .*

**Definition** **2** ( UniformlyUltimatelyBounded [24]). *The time function x(t) is said to be uniformly ultimately bounded (UUB) on a compact set $\Omega_{x}$ , if, for all $x\left(t_{0}\right)=x_{0}\in \Omega_{x}$ , there exists a $\delta_{2}>0$ and a number $T(\delta_{2},x_{0})$ such that $∥x\left(t\right)∥<\delta_{2}$ for all $t\geq t_{0}+T$ .*

### 2.2. Problem Formulation

Consider the nonlinear continuous-time (NCT) systems with disturbances described by the following dynamics:

(1) $$\begin{array}{cc} \; & \dot{x}\left(t\right)=f\left(x\right)+g\left(x\right)u\left(t\right)+G\left(x\right)d\left(t\right), \end{array}$$

where $x\left(t\right)\in R^{n}$ and $u\left(t\right)\in R^{m}$ denote the system state and control input, respectively. $d\left(t\right)\in R^{q}$ represents the external disturbance. The terms $f\left(x\right)\in R^{n}$ , $g\left(x\right)\in R^{n\times m}$ , and $G\left(x\right)\in R^{n\times q}$ are the drift dynamics, input dynamics, and disturbance injection dynamics, respectively. In this study, f(x) , g(x) , and G(x) are assumed to be unknown. Furthermore, it is assumed that f(x) , g(x) , and G(x) are Lipschitz continuous with f(0)=0 , and that the system (1) is stabilizing and controllable.

The goal of this study is to solve an $H_{\infty }$ control problem for the system (1). This problem can be equivalently transformed into a two-player zero-sum game, where the control input u(t) acts as the minimizing player and the disturbance d(t) acts as the maximizing player. The solution to the $H_{\infty }$ control problem corresponds to a saddle point in the game, which stabilizes the equilibrium of the two-player zero-sum game.

Define the infinite-horizon performance index function as

(2) $$\begin{array}{c} V\left(x,u,d\right)=\int_{t}^{\infty }\left(x^{T}Qx+u^{T}Ru-\kappa^{2}d^{T}d\right)d\tau , \end{array}$$

where κ>0 , V(0)=0 , and *Q* and *R* are symmetric positive-definite matrices with appropriate dimensions. Let $u^{★}$ be the optimal control input and $d^{★}$ be the worst disturbance. Our objective is to find the saddle point $\left(u^{★},d^{★}\right)$ that optimizes the performance index (2), which can be more precisely clarified by the following inequality:

(3) $$\begin{array}{c} V\left(u^{★},d\right)\leq V\left(u^{★},d^{★}\right)\leq V\left(u,d^{★}\right). \end{array}$$

We then define the optimal performance index function $V^{★}$ as follows:

(4) $$\begin{array}{c} V^{★}\left(x,u,d\right)\;=\;\min_{u}\max_{d}\int_{t}^{\infty }\left(x^{T}Qx+u^{T}Ru-\kappa^{2}d^{T}d\right)d\tau . \end{array}$$

The Hamiltonian of system (1) can be written as  

(5) $$\begin{array}{cc} H(V_{x},x,u,d)= & V_{x}^{T}\left[f\left(x\right)+g\left(x\right)u+G\left(x\right)d\right]+x^{T}Qx+u^{T}Ru-\kappa^{2}d^{T}d, \end{array}$$

where $V_{x}=\partial V/\partial x\in R^{n}$ . The Hamilton–Jacobi–Isaacs (HJI) equation related to this game has the form

(6) $$\begin{array}{c} \min_{u}\max_{d}\;\;H\left(V_{x}^{★},x,u,d\right)=0, \end{array}$$

where $V_{x}^{★}=\partial V^{★}/\partial x\in R^{n}$ . Based on the stationarity condition, the $H_{\infty }$ control pair $\left(u^{★},d^{★}\right)$ for (1) has the following form:

(7) $$\begin{array}{c} u^{★}=-\frac{1}{2}R^{-1}g^{T}\left(x\right)V_{x}^{★}\left(x\right), \end{array}$$

(8) $$\begin{array}{c} d^{★}=\frac{1}{2\kappa^{2}}G^{T}\left(x\right)V_{x}^{★}\left(x\right). \end{array}$$

Thus, according to (7) and (8), the HJI Equation (6) can be rewritten as

(9) $$\begin{array}{cc} x^{T}Qx & +V_{x}^{★T}f\left(x\right)-\frac{1}{4}V_{x}^{★T}g\left(x\right)R^{-1}g^{T}\left(x\right)V_{x}^{★}+\frac{1}{4\kappa^{2}}V_{x}^{★T}G\left(x\right)G^{T}\left(x\right)V_{x}^{★}=0. \end{array}$$

**Figure 1:**
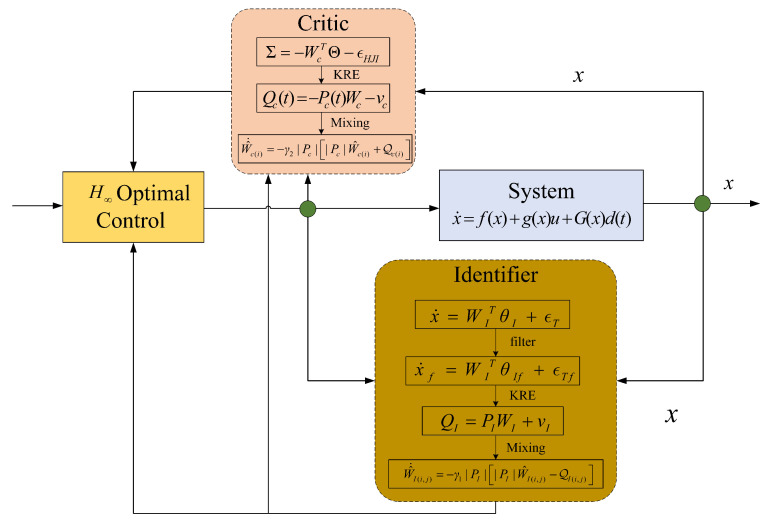
Schematic of the proposed control system.

Indeed, the HJI Equation (9) represents a highly nonlinear partial differential equation (PDE) and requires complete system information for its resolution. To address these challenges, a new IC framework with relaxed PE conditions will be proposed in the following sections. Furthermore, new adaptive update laws for the identifier and critic NNs are provided with the help of the KREM technique. The block diagram of the proposed control system is shown in Figure 1, and detailed theoretical analysis will be presented in subsequent sections.

## 3. System Identifier Design with Relaxed PE Condition

In this section, an NN-based identifier is utilized to reconstruct the unknown system dynamics in (1). The KREM technique is introduced to adjust the identifier weights under relaxed PE conditions. We assume that the unknown system dynamics f(x) , g(x) , and G(x) in (1) are continuous functions defined on compact sets. The NN-based identifier is designed as follows:

(10) $$f\left(x\right)=W_{f}\theta_{f}\left(x\right)+\epsilon_{f},$$

(11) $$g\left(x\right)=W_{g}\theta_{g}\left(x\right)+\epsilon_{g},$$

(12) $$G\left(x\right)=W_{G}\theta_{G}\left(x\right)+\epsilon_{G},$$

where $W_{f}\in R^{n\times d_{f}}$ , $W_{g}\in R^{n\times d_{g}}$ and $W_{G}\in R^{n\times d_{G}}$ are the ideal NN weights; $\theta_{f}\left(x\right)\in R^{d_{f}}$ , $\theta_{g}\left(x\right)\in R^{d_{g}\times m}$ and $\theta_{G}\left(x\right)\in R^{d_{G}\times q}$ are the basis functions; and $\epsilon_{f}\in R^{n}$ , $\epsilon_{g}\in R^{n\times m}$ and $\epsilon_{G}\in R^{n\times q}$ are the reconstruction errors. Then, according to the Weierstrass theorem and the statements in [10], the approximation errors $\epsilon_{f}$ , $\epsilon_{g}$ , and $\epsilon_{G}$ can be shown to approach zero as the number of NN neurons $d_{f}$ , $d_{g}$ , and $d_{G}$ increases to infinity.

Before proceeding, it is essential to establish the following underlying assumption.

**Assumption** **1.**

*(1)*  *The basis functions $\theta_{f}\left(x\right)$ , $\theta_{g}\left(x\right)$ and $\theta_{G}\left(x\right)$ are bounded, that is, $∥\theta_{f}\left(x\right)∥\leq b_{\theta_{f}}$ , $∥\theta_{g}\left(x\right)∥\leq b_{\theta_{g}}$ , $∥\theta_{G}\left(x\right)∥\leq b_{\theta_{G}}$ , respectively.*
*(2)*  *The reconstruction errors $\varepsilon_{f}$ , $\varepsilon_{g}$ and $\varepsilon_{G}$ are bounded, that is, $∥\varepsilon_{f}∥\leq b_{\varepsilon_{f}}$ , $∥\varepsilon_{g}∥\leq b_{\varepsilon_{g}}$ , $∥\varepsilon_{G}∥\leq b_{\varepsilon_{G}}$ , respectively.*

Using (10)–(12), the system (1) can be rewritten as

(13) $$\dot{x}=W_{I}^{T}\theta_{I}\left(x,u\right)+\epsilon_{T},$$

where $W_{I}={\left[W_{f},W_{g},W_{G}\right]}^{T}\in R^{d\times n}$ is the augmented weight matrix with $d=d_{f}+d_{g}+d_{G}$ , and $\theta_{I}\left(x,u\right)={\left[\theta_{f}^{T}\left(x\right),u^{T}\theta_{g}^{T}\left(x\right),d^{T}\theta_{G}^{T}\left(x\right)\right]}^{T}\in R^{d}$ is the augmented regressor vector. $\epsilon_{T}=\epsilon_{f}+\epsilon_{g}u+\epsilon_{G}d\in R^{n}$ is the model approximation error.

Note that $\dot{x}$ and $W_{I}$ are unknown. Therefore, we define the filtered variables $x_{f}$ and $\theta_{If}$ as

(14) $$\left\{\begin{array}{c} \rho {\dot{x}}_{f}+x_{f}=x,\;\;\;\;x_{f}\left(0\right)=0\\ \rho {\dot{\theta }}_{If}+\theta_{If}=\theta_{I},\;\;\;\theta_{If}\left(0\right)=0\; \end{array}\right.$$

where ρ∈R>0 is the filter coefficient. From Equations (13) and (14), we can deduce that

(15) $${\dot{x}}_{f}=\frac{x-x_{f}}{\rho }=W_{I}^{T}\theta_{If}+\epsilon_{Tf},$$

where $\epsilon_{Tf}$ denotes the filtered version of $\epsilon_{T}$ as $\rho {\dot{\epsilon }}_{Tf}+\epsilon_{Tf}=\epsilon_{T}$ . Clearly, (15) is a linear regression equation (LRE), where ${\dot{x}}_{f}$ and $\theta_{If}$ can be calculated from (14). In the following, we describe how the KREM technique is applied to estimate $W_{I}$ by using the measured information ${\dot{x}}_{f}$ and $\theta_{If}$ .

To approximate the unknown weights $W_{I}$ in (15) such that the estimated weights ${\hat{W}}_{I}$ converge to their true values under a relaxed PE condition, we aim to construct an extended LRE (E-LRE) based on (15). We define the matrices $P_{I}\in R^{d\times d}$ and $Q_{I}\in R^{d\times n}$ as follows:

(16) $$\begin{array}{c} \left\{ \begin{array}{c} P_{I}=H_{I}\left[\theta_{If}\theta_{If}^{T}\right],\;P_{I}\left(0\right)=0\\ Q_{I}=H_{I}\left[\theta_{If}{\left(\frac{x-x_{f}}{\rho }\right)}^{T}\right],\;Q_{I}\left(0\right)=0 \end{array}\right. \end{array}$$

where

$$\begin{array}{c} H_{I}=\frac{1}{p+l_{I}}\left[s\right]\left(t\right) \end{array}$$

with p=d/dt , $l_{I}>0$ is a forgetting factor. From (16), we can derive its solution as

(17) $$\left\{ \begin{array}{c} P_{I}=\int_{0}^{t}e^{-l_{I}\left(t-\tau \right)}\theta_{If}\left(\tau \right)\theta_{If}^{T}\left(\tau \right)d\left(\tau \right),\\ Q_{I}=\int_{0}^{t}e^{-l_{I}\left(t-\tau \right)}\theta_{If}\left(\tau \right){\left(\frac{x\left(\tau \right)-x_{f}\left(\tau \right)}{\rho }\right)}^{T}d\left(\tau \right)\;. \end{array}\right.$$

Note that it can be verified that $P_{I}$ and $Q_{I}$ are bounded for any given bounded $\theta_{I}$ and *x* due to the appropriate choice of $l_{I}$ . Thus, an E-LRE is obtained

(18) $$Q_{I}\left(t\right)=P_{I}\left(t\right)W_{I}+v_{I},$$

where $v_{I}=\int_{0}^{t}e^{-l_{I}\left(t-\tau \right)}\theta_{If}\left(\tau \right)\epsilon_{Tf}^{T}\left(\tau \right)d\left(\tau \right)$ .

To construct an identifier weight error dynamics that achieves better convergence properties, we define the variables $Q_{I}\left(t\right)\in R^{d\times n}$ , $P_{I}\in R^{d\times d}$ , and $V_{I}\in R^{d\times n}$ as follows:

(19) $$\begin{array}{c} \left\{ \begin{array}{c} Q_{I}=P_{I}^{*}Q_{I},\\ P_{I}=P_{I}^{*}P_{I},\\ V_{I}=P_{I}^{*}v_{I}. \end{array}\right. \end{array}$$

Then Equation (18) becomes

(20) $$Q_{I}\left(t\right)=P_{I}\left(t\right)W_{I}+V_{I}.$$

Note that for any square matrix $M\in R^{q\times q}$ , we have $M^{*}M=\left|M\right|I_{q}$ , even if *M* is not full rank. Thus, $P_{I}=\left|P_{I}\right|I_{d}\in R^{d\times d}$ . Moreover, $P_{I}$ is a scalar diagonal matrix, where (20) can be decoupled into a series of scalar LREs:

(21) $${Q_{I}}_{\left(i,j\right)}\left(t\right)=\left|P_{I}\right|\left(t\right){W_{I}}_{\left(i,j\right)}+V_{I(i,j)},i=1,\ldots ,d,j=1,\ldots ,n,$$

where ${Q_{I}}_{\left(i,j\right)}$ and ${W_{I}}_{\left(i,j\right)}$ indicate the *i*th row and *j*th column of $Q_{I}$ and $W_{I}$ , respectively.

Then, the estimation algorithm for the unknown identifier NN weights can be designed based on (21) as follows:

(22) $${\dot{\hat{W}}}_{I(i,j)}=-\gamma_{1}|P_{I}\left|[\right|P_{I}\left|{\hat{W}}_{I(i,j)}-Q_{I(i,j)}\right],$$

where $\gamma_{1}\in R>0$ presents the adaptive learning gain.

The convergence of identifier (22) can be given as follows.

**Theorem** **1.** *Consider the system (13) with the online update law (22); if $|P_{I}|\;\in PE$ , then*

*(i)*  *for $\epsilon_{T}=0$ , the estimator error ${\tilde{W}}_{I(i,j)}$ converges to zero exponentially;*
*(ii)*  *for $\epsilon_{T}\neq 0$ , the estimator error ${\tilde{W}}_{I(i,j)}$ converges to a compact set around zero.*

**Proof.**  If $|P_{I}|\in PE$ , according to Definition 1 we have $\int_{t}^{t+T}|P_{I}{|}^{2}dr\geq \delta_{I}>0$ . Defining the estimation error ${\tilde{W}}_{I(i,j)}={\hat{W}}_{I(i,j)}-W_{I(i,j)}$ , i=1,…,d , j=1,…,n . Due to (21) and (22), the identifier weight error dynamics can be obtained

(23) $${\dot{\tilde{W}}}_{I(i,j)}=-\gamma_{1}|P_{I}{|}^{2}{\tilde{W}}_{I(i,j)}+\gamma_{1}\left|P_{I}\right|V_{I(i,j)}.$$

Considering the Lyapunov function $V_{I}=0.5\gamma_{1}^{-1}{\tilde{W}}_{I(i,j)}^{2}$ , the derivation of $V_{I}$ can be calculated as

(24) $$\begin{array}{cc} {\dot{V}}_{I} & =\frac{1}{\gamma_{1}}{\tilde{W}}_{I(i,j)}{\dot{\tilde{W}}}_{I(i,j)}\\ \; & =-|P_{I}{|}^{2}{\tilde{W}}_{I(i,j)}^{2}+\left|P_{I}\right|{\tilde{W}}_{I(i,j)}V_{I(i,j)}. \end{array}$$

In fact, when $\epsilon_{T}=0$ , (24) can be rewritten as

(25) $$\begin{array}{c} {\dot{V}}_{I}=-{\left|P_{I}\right|}^{2}{\tilde{W}}_{I(i,j)}^{2}<-\mu_{I}V_{I}, \end{array}$$

where $\mu_{I}=2\gamma_{1}\delta_{I}>0$ . According to the Lyapunov theorem, the weight estimation error ${\tilde{W}}_{I(i,j)}$ exponentially converges to zero.

When $\epsilon_{T}\neq 0$ , (24) can be further presented as

(26) $$\begin{array}{cc} {\dot{V}}_{I} & =-|P_{I}{|}^{2}{\tilde{W}}_{I(i,j)}^{2}+\left|P_{I}\right|{\tilde{W}}_{I(i,j)}V_{I(i,j)}\\ \; & =-\left[\right|P_{I}{|}^{2}{\tilde{W}}_{I(i,j)}-|P_{I}\left|V_{I(i,j)}\right]{\tilde{W}}_{I(i,j)}. \end{array}$$

According to Assumption 1, $|P_{I}|V_{I(i,j)}$ is bounded, denoted as $∥|P_{I}|V_{I(i,j)}∥<b_{P_{I}V_{I}}$ . Then,  

(27) $$\begin{array}{cc} {\dot{V}}_{I} & \leq -\left[\right|P_{I}{|}^{2}\left|\right|{\tilde{W}}_{I(i,j)}\left|\right|-b_{P_{I}V_{I}}]||{\tilde{W}}_{I(i,j)}\left|\right|. \end{array}$$

According to the extended Lyapunov theorem, the estimation error ${\tilde{W}}_{I(i,j)}$ uniformly ultimately converges to a compact set $\left\{{\tilde{W}}_{I(i,j)}|||{\tilde{W}}_{I(i,j)}||\leq b_{P_{I}V_{I}}/p_{I}^{2}\right\}$ .    □

**Remark** **1.** *In [12], the update law for the unknown weight $W_{I}$ was designed based on (18), while the PE condition (i.e., $\theta_{I}\in PE$ ) was required to ensure convergence. However, satisfying the PE condition is generally challenging. In Theorem 1, we provide a new convergence condition $|P_{I}|\in PE$ . Notably, this new condition is significantly superior to the conventional PE condition for two reasons. (1) We theoretically prove that $|P_{I}|\in PE$ is much weaker than $\theta_{I}\in PE$ , as detailed in Section 5. (2) $|P_{I}|$ is directly related to the determinant of the matrix $P_{I}\left(t\right)$ . Therefore, checking $|P_{I}|\in PE$ online becomes feasible by calculating the determinant of $P_{I}\left(t\right)$ . In contrast, assessing the standard PE condition directly online is not possible [18,19,23].*

Based on the above analysis, the unknown information f(x) , g(x) , and G(x) can be estimated using (13) and (22). This allows for the reconstruction of the completely unknown system dynamics. In order to obtain the optimal $H_{\infty }$ control pair, the critic NN will be introduced to learn the solution of the HJB equation in the subsequent section.

## 4. Critic NN Design for $H_{\infty }$ Control under Relaxed PE Condition

In this section, the performance index will be approximated via a critic NN to obtain the optimal $H_{\infty }$ control pair. The KREM algorithm will be continually utilized to design the update law of critic NN under the relaxed PE condition. Firstly, based on the above identifier, the system (1) can be represented as

(28) $$\dot{x}={\hat{W}}_{f}\theta_{f}\left(x\right)+{\hat{W}}_{g}\theta_{g}\left(x\right)u+{\hat{W}}_{G}\theta_{G}\left(x\right)d\left(t\right)+\epsilon_{I}+\epsilon_{T},$$

where ${\hat{W}}_{f}$ , ${\hat{W}}_{g}$ and ${\hat{W}}_{G}$ are the estimated values of $W_{f}$ , $W_{g}$ and $W_{G}$ , respectively. $\epsilon_{I}={\tilde{W}}_{I}\theta_{I}$ denotes the identifier error. And, the Hamiltonian (5) can be further written as

(29) $$\begin{array}{cc} H= & V_{x}^{T}\left[{\hat{W}}_{f}\theta_{f}\left(x\right)+{\hat{W}}_{g}\theta_{g}\left(x\right)u+{\hat{W}}_{G}\theta_{G}\left(x\right)d\left(t\right)+\epsilon_{I}+\epsilon_{T}\right]+x^{T}Qx+u^{T}Ru-\kappa^{2}d^{T}d. \end{array}$$

Then, the HJI Equation (6) becomes

(30) $$\begin{array}{cc} 0 & =\min_{u}\max_{d}\left[H\left(V_{x}^{★},x,u^{★},d^{★}\right)\right]\\ & =V_{x}^{★T}\left[{\hat{W}}_{f}\theta_{f}\left(x\right)+{\hat{W}}_{g}\theta_{g}\left(x\right)u^{★}+{\hat{W}}_{G}\theta_{G}\left(x\right)d^{★}\left(t\right)+\epsilon_{I}+\epsilon_{T}\right]+x^{T}Qx+u^{★T}Ru^{★}-\kappa^{2}d^{★T}d^{★}. \end{array}$$

Therefore, based on (30), the $H_{\infty }$ control pair $\left(u^{★},d^{★}\right)$ for the estimated system (28) can be expressed as follows:

(31) $$\begin{array}{c} u^{★}=-\frac{1}{2}R^{-1}{\left[{\hat{W}}_{g}\theta_{g}\right]}^{T}V_{x}^{★}, \end{array}$$

(32) $$\begin{array}{c} d^{★}=\frac{1}{2\kappa^{2}}{\left[{\hat{W}}_{G}\theta_{G}\right]}^{T}V_{x}^{★}\left(x\right). \end{array}$$

Since the HJI Equation (30) is a nonlinear PDE, similar to (6), we utilize a critic NN to estimate $V^{★}\left(x\right)$ and its gradient $V_{x}^{★}\left(x\right)$ as follows:

(33) $$V^{★}\left(x\right)=W_{c}^{T}\theta_{c}\left(x\right)+\epsilon_{v},$$

(34) $$V_{x}^{★}\left(x\right)=\nabla \theta_{c}^{T}\left(x\right)W_{c}+\nabla \epsilon_{v},$$

where $W_{c}\in R^{l}$ is the unknown constant weight. $\theta_{c}\left(x\right)\in R^{l}$ represents the independent basis function with $\nabla \theta_{c}\left(x\right)=\partial \theta_{c}/\partial x$ . *l* is the number of neurons. The approximation error is presented as $\epsilon_{v}$ with $\nabla \epsilon_{v}=\partial \epsilon_{v}/\partial x$ . Note that as the number of independent basis functions increases, both the approximation errors and their gradients can approach zero.

Before proceeding, the following assumption is needed.

**Assumption** **2.**

*(1)*  *The ideal critic NN’s weight $W_{c}$ is bounded, that is, $∥W_{c}∥<b_{W_{c}}$ .*
*(2)*  *The basis functions $\theta_{c}\left(x\right)$ and its gradients $\nabla \theta_{c}\left(x\right)$ are bounded, that is, $∥\theta_{c}∥\leq b_{\theta_{c}}$ , $∥\nabla \theta_{c}∥\leq b_{\nabla \theta_{c}}$ .*
*(3)*  *The approximator reconstruction error $\epsilon_{v}$ and its gradients $\nabla \epsilon_{v}$ are bounded, that is, $∥\epsilon_{v}∥\leq b_{\epsilon_{v}}$ , $∥\nabla \epsilon_{v}∥\leq b_{\nabla \epsilon_{v}}$ .*

Since the ideal critic NN weights $W_{c}$ are unknown, take ${\hat{W}}_{c}$ as the estimated value of $W_{c}$ and $\hat{V}$ as the estimated value of *V*, where the practical critic NN is given by

(35) $$\begin{array}{cc} \; & \;\;\hat{V}\left(x\right)={\hat{W}}_{c}^{T}\theta_{c}\left(x\right),\\ \; & {\hat{V}}_{x}\left(x\right)=\nabla \theta_{c}^{T}\left(x\right){\hat{W}}_{c}. \end{array}$$

The estimated $H_{\infty }$ control pair $\hat{u}$ and $\hat{d}$ can be obtained as

(36) $$\begin{array}{c} \hat{u}=-\frac{1}{2}R^{-1}{\left[{\hat{W}}_{g}\theta_{g}\right]}^{T}{\hat{V}}_{x}=-\frac{1}{2}R^{-1}{\left[{\hat{W}}_{g}\theta_{g}\right]}^{T}\nabla \theta_{c}^{T}{\hat{W}}_{c}, \end{array}$$

(37) $$\begin{array}{c} \hat{d}=\frac{1}{2\kappa^{2}}{\left[{\hat{W}}_{G}\theta_{G}\right]}^{T}{\hat{V}}_{x}=\frac{1}{2\kappa^{2}}{\left[{\hat{W}}_{G}\theta_{G}\right]}^{T}\nabla \theta_{c}^{T}{\hat{W}}_{c}. \end{array}$$

To online estimate the unknown weights of the critic NN using KREM technology, we aim to construct a linear equation according to (30) and (34) as

(38) $$\begin{array}{cc} \; & \epsilon_{HJI}+x^{T}Qx+{\hat{u}}^{T}R\hat{u}-\kappa^{2}{\hat{d}}^{T}\hat{d}+W_{c}^{T}\nabla \theta_{c}{\hat{W}}_{f}\theta_{f}\left(x\right)\\ \; & \;\;\;\;\;\;\;\;\;\;\;\;\;\;\;\;+W_{c}^{T}\nabla \theta_{c}{\hat{W}}_{g}\theta_{g}\left(x\right)\hat{u}+W_{c}^{T}\nabla \theta_{c}{\hat{W}}_{G}\theta_{G}\left(x\right)\hat{d}=0, \end{array}$$

where $\epsilon_{HJI}=W_{c}^{T}\nabla \theta_{c}\left(\epsilon_{I}+\epsilon_{T}\right)+\nabla \epsilon_{v}^{T}\left({\hat{W}}_{f}\theta_{f}+{\hat{W}}_{g}\theta_{g}\hat{u}+{\hat{W}}_{G}\theta_{G}\hat{d}+\epsilon_{I}+\epsilon_{T}\right)$ is a bounded residual HJI equation error. Let $\Theta =\nabla \theta_{c}\left[{\hat{W}}_{f}\theta_{f}+{\hat{W}}_{g}\theta_{g}\hat{u}+{\hat{W}}_{G}\theta_{G}\hat{d}\right]$ and $\Sigma =x^{T}Qx+{\hat{u}}^{T}R\hat{u}-\kappa^{2}{\hat{d}}^{T}\hat{d}$ , where a linear equation is obtained as follows:

(39) $$\begin{array}{c} \Sigma =-W_{c}^{T}\Theta -\epsilon_{HJI}. \end{array}$$

Similar to the previous section, we define the filtered regressor matrix $P_{c}\in R^{l\times l}$ and the vector $Q_{c}\in R^{l}$ as follows:

(40) $$\begin{array}{c} \left\{\begin{array}{c} P_{c}=H_{c}\left[\Theta \Theta^{T}\right],\;P_{c}\left(0\right)=0\\ Q_{c}=H_{c}\left[\Theta \Sigma \right],\;Q_{c}\left(0\right)=0 \end{array}\right. \end{array}$$

where

$$H_{c}=\frac{1}{p+l_{c}}\left[s\right]\left(t\right),$$

and $l_{c}>0$ is the forgetting factor. Then, the solution of (40) can be deduced as

(41) $$\begin{array}{c} \left\{\begin{array}{c} P_{c}=\int_{0}^{t}e^{-l_{c}\left(t-\tau \right)}\Theta \Theta^{T}d\tau ,\\ Q_{c}=\int_{0}^{t}e^{-l_{c}\left(t-\tau \right)}\Theta \Sigma d\tau . \end{array}\right. \end{array}$$

From (39) and (41), an E-LRE related to $P_{c}$ and $Q_{c}$ is obtained

(42) $$Q_{c}\left(t\right)=-P_{c}\left(t\right)W_{c}-v_{c},$$

where $v_{c}=\int_{0}^{t}e^{-l_{c}\left(t-\tau \right)}\Theta \left(\tau \right)\epsilon_{HJI}^{T}\left(\tau \right)d\tau$ is bounded. To estimate the unknown parameter $W_{c}$ in (42) under a relaxed PE condition, define the variables $Q_{c}\left(t\right)\in R^{l}$ , $P_{c}\in R^{l\times l}$ , and $V_{c}\in R^{l}$ as  

(43) $$\begin{array}{c} \left\{\begin{array}{c} Q_{c}=P_{c}^{*}Q_{c},\\ P_{c}=P_{c}^{*}P_{c},\\ V_{c}=P_{c}^{*}v_{c}. \end{array}\right. \end{array}$$

Then Equation (42) becomes

(44) $$Q_{c}\left(t\right)=-P_{c}\left(t\right)W_{c}-V_{c}.$$

Note that $P_{c}=\left|P_{c}\right|I_{l}$ . Since $P_{c}$ is a scalar matrix, a series of scalar LREs is obtained as

(45) $${Q_{c}}_{\left(i\right)}\left(t\right)=-\left|P_{c}\right|\left(t\right)W_{c(i)}-V_{c(i)},i=1,\cdots ,l,$$

where ${Q_{c}}_{\left(i\right)}$ , $W_{c(i)}$ and $V_{c(i)}$ indicate the ith rows of $Q_{c}$ , $W_{c}$ , and $V_{c}$ , respectively.

Driven by the parameter error based on (45), the critic unknown weight $W_{c(i)}$ is designed as

(46) $${\dot{\hat{W}}}_{c(i)}=-\gamma_{2}|P_{c}\left|[\right|P_{c}\left|{\hat{W}}_{c(i)}+Q_{c(i)}\right],$$

where $\gamma_{2}\in R>0$ presents the adaptive learning gain.

The convergence condition for the proposed critic NN adaptive law is provided in Theorem 2.

**Theorem** **2.** *For adaptive law (46) of critic NN with the regressor matrix $P_{c}$ in (44); if $|P_{c}|\;\in PE$ , then*

*(i)*  *for $\epsilon_{HJI}=0$ , the estimator error ${\tilde{W}}_{c(i)}$ converges to zero exponentially;*
*(ii)*  *for $\epsilon_{HJI}\neq 0$ , the estimator error ${\tilde{W}}_{c(i)}$ converges to a compact set around zero;*

**Proof.**  Defining the estimation error ${\tilde{W}}_{c(i)}={\hat{W}}_{c(i)}-W_{c(i)}$ , i=1,…,l . The proofs presented in Theorem 1 can be extended to establish similar results in the current context. Note that the Lyapunov function $V_{c}$ here is chosen as $0.5\gamma_{2}^{-1}{\tilde{W}}_{c(i)}^{2}$ .    □

**Remark** **2.** *According to Theorem 2, a new convergence condition for the estimation error of the critic neural network weights, denoted as ${\tilde{W}}_{c}$ , is provided. This condition does not rely on the conventional parameter estimation (PE) condition, i.e., Θ∈PE . In this paper, the additional exploration signal is not required to guarantee Θ∈PE . Instead, the satisfaction of $|P_{c}|\in PE$ can be achieved by adjusting the forgetting factor $l_{c}$ . It is worth noting that the new convergence condition is associated with the matrix $P_{c}$ , and it can be verified online by calculating the determinant of $P_{c}$ . The proof of the weak PE property for the new convergence condition will be presented in the following section.*

**Remark** **3.** *The convergence analysis of ${\tilde{W}}_{I(i,j)}$ and ${\tilde{W}}_{c(i)}$ are provided in Theorem 1 and Theorem 2, respectively. In fact, we can derive the convergence of ${\tilde{W}}_{I}$ and ${\tilde{W}}_{c}$ using simple matrix operations, which will be omitted in this paper.*

Till now, the identifier–critic learning-based framework for $H_{\infty }$ optimal control under the relaxed PE condition is given. For clarity, the design details of the proposed method are shown in Algorithm 1, which can be considered the pseudocode for the simulation part.

**Algorithm 1** Identifier–critic learning-based 
$H_{\infty }$
 optimal control algorithm1:**Initialization**2:Initialize system parameters: x(0) , *Q*, *R* and running time *T*;3:Set the identifier and critic filter operators: $H_{I}$ and $H_{c}$ ;4:Set the basis functions of identifier and critic NNs: $\theta_{I}\left(x,u\right)$ and $\theta_{c}\left(x\right)$ ;5:Initialize and set the filter operator parameters: ρ , $l_{I}$ , $l_{c}$ , $x_{f}\left(0\right)=0$ , $\theta_{If}\left(0\right)=0$ and $\epsilon_{If}\left(0\right)=0$ ;6:Initialize identifier NNs parameters: $\gamma_{1}>0$ , ${\hat{W}}_{I}^{initial}\in \left(0,1\right]$ ;7:Initialize critic NNs parameters: $\gamma_{2}>0$ , ${\hat{W}}_{c}^{initial}\in \left(0,1\right]$ ;8:Initialize the control pair by (36) and (37) ;9:**while** t≤T **do**10:   Calculate the filter processing of the identifier NNs by (14) ;11:   Calculate the dynamic regressor extension (DRE) of the identifier NNs by (15) ;12:   Calculate the regressor “mixing” of the identifier NNs by (18) ;13:   Update the weight parameters of the identifier NNs ${\hat{W}}_{I(i,j)}$ by (20) ;

$${\dot{\hat{W}}}_{I(i,j)}=-\gamma_{1}|P_{I}\left|[\right|P_{I}\left|{\hat{W}}_{I(i,j)}-Q_{I(i,j)}\right];$$
14:   Compute the approximated HJB equation by (39) ;15:   Calculate the dynamic regressor extension (DRE) of the critic NNs by (40) ;16:   Calculate the regressor “mixing” of the critic NNs by (42) ;17:   Update the weight parameters of the critic NNs ${\hat{W}}_{c(i)}$ by (46) ;

$${\dot{\hat{W}}}_{c(i)}=-\gamma_{2}|P_{c}\left|[\right|P_{c}\left|{\hat{W}}_{c(i)}+Q_{c(i)}\right];$$
18:   Update the control pair by (36) and (37) ;19:   Update the system states *x* by (28) ;20:**end while**


## 5. Stability and Convergence Analysis

In this section, we present the main results, which include the theoretical analysis of weak PE properties under new convergence conditions proposed in Theorem 1 and Theorem 2. Furthermore, we provide a stability result for the closed-loop system under the proposed online learning optimal control method.

To facilitate the analysis, the following assumption is made.

**Assumption** **3.** *The system dynamics in (1) satisfy $∥f(x)∥\leq b_{f}∥x∥$ , $∥g(x)∥\leq b_{g}$ and $∥G(x)∥\leq b_{G}$ , where $b_{f}>0$ , $b_{g}>0$ and $b_{G}>0$ .*

### 5.1. Weak PE Properties of New Convergence Conditions

As shown in Theorem 1, Theorem 2 and Remark 3, the convergence of ${\tilde{W}}_{I}$ and ${\tilde{W}}_{c}$ is established without the restrictive PE condition, i.e., $\theta_{I}\in PE$ and Θ∈PE . These new convergence conditions can be easily checked online, as mentioned in Remark 1 and Remark 2. Furthermore, we will analyze the superiority of the new convergence conditions compared to the conventional PE condition from a theoretical standpoint.

**Theorem** **3.** *Consider the system (13) with the online identifier NN adaptive law (22) and critic NN adaptive law (46),*

*(i)*  *The convergence condition of estimation error ${\tilde{W}}_{I}$ in Theorem 1, that is, $|P_{c}|\in PE$ , is weaker than $\theta_{I}\in PE$ in the following precise sense*
(47) $$\theta_{I}\left(t\right)\in PE\Rightarrow \left|P_{c}\right|\in PE,$$
(48) $$|P_{c}|\in PE⇏\theta_{I}\left(t\right)\in PE;$$
*(ii)*  *The convergence condition of estimation error ${\tilde{W}}_{c}$ in Theorem 2, that is, $|P_{c}|\in PE$ , is weaker than Θ∈PE in the following precise sense*
(49) $$\Theta \in PE\Rightarrow |P_{c}|\in PE,$$
(50) $$|P_{c}|\in PE⇏\Theta \in PE.$$

**Proof.**  For (i) , suppose that $\theta_{I}\left(t\right)$ in (13) is PE, indicating that $\theta_{If}\left(t\right)\in PE$ [25]. From Definition 1, we have

(51) $$\begin{array}{cc} \; & \int_{t}^{t+\tau }\theta_{If}\left(r\right)\theta_{If}^{T}\left(r\right)dr\geq \delta I\Leftrightarrow \;\;\int_{t-\tau }^{t}\theta_{If}\left(r\right)\theta_{If}^{T}\left(r\right)dr\geq \delta I\;\;\mathrm{for}\;\;t>\tau >0. \end{array}$$

Moreover, since $e^{-\beta_{I}\left(t-r\right)}\geq e^{-\beta_{I}\tau }>0$ with r∈[t−τ,t] , the following inequality holds

(52) $$\begin{array}{cc} \; & \int_{t-\tau }^{t}e^{-\beta_{I}\left(t-r\right)}\theta_{If}^{T}\left(r\right)\theta_{If}\left(r\right)dr\geq \int_{t-\tau }^{t}e^{-\beta_{I}\tau }\theta_{If}^{T}\left(r\right)\theta_{If}\left(r\right)dr\geq e^{-\beta_{I}\tau }\delta I. \end{array}$$

Furthermore, for t>τ>0 , we also have

(53) $$\begin{array}{cc} \; & \int_{0}^{t}e^{-\beta_{I}\left(t-r\right)}\theta_{If}^{T}\left(r\right)\theta_{If}\left(r\right)dr>\int_{t-\tau }^{t}e^{-\beta_{I}\left(t-r\right)}\theta_{If}^{T}\left(r\right)\theta_{If}\left(r\right)dr. \end{array}$$

From (17), (52) and (53), we conclude that

(54) $$\begin{array}{cc} P_{I} & =\int_{0}^{t}e^{-\beta_{I}\left(t-r\right)}\theta_{If}^{T}\left(r\right)\theta_{If}\left(r\right)dr>e^{-\beta_{I}\tau }\int_{t-\tau }^{t}\theta_{If}^{T}\left(r\right)\theta_{If}\left(r\right)dr\geq e^{-\beta_{I}\tau }\delta I. \end{array}$$

Hence, the matrix $P_{I}$ in (16) is positive definite, that is, $\lambda_{i}\left(P_{I}\right)>0$ , i=1,…,d . Considering that the determinant of a matrix is equal to the product of all its eigenvalues, that is, $|P_{I}|=\lambda_{1}\left(P_{I}\right)\lambda_{2}\left(P_{I}\right)\ldots \lambda_{d}\left(P_{I}\right)$ , we obtain $\lambda_{i}\left(P_{I}\right)>0\Rightarrow \prod_{i=1}^{d}\lambda_{i}\left(P_{I}\right)>0\Rightarrow \left|P_{I}\right|>0$ . Thus, (47) is true.

The proof of (48) is established by the following:

(55) $$\begin{array}{cc} |P_{I}|\in PE & \Leftrightarrow \int_{0}^{t}|P_{I}{|}^{2}\left(\tau \right)d\tau >0\\ \; & \Leftrightarrow \int_{0}^{t}\prod_{i=1}^{d}\lambda_{i}^{2}\left(P_{I}\right)>0\\ \; & ⇏\lambda_{i}\left(P_{I}\right)>0,i=1,\ldots ,d\\ \; & \Leftrightarrow P_{I}>0\\ \; & \Leftrightarrow P_{I}\in PE. \end{array}$$

For *(ii)*, the proof process can be referred to in *(i)*. This finishes the proof. □

### 5.2. Stability and Convergence Analysis

The stability result for the closed-loop system under the proposed online learning optimal control method will be presented in the following theorem.

**Theorem** **4.** *Let Assumptions 1 and 2 hold. Considering system (1) with the identifier weight tuning law given by (22), the $H_{\infty }$ control pair are computed by (36) and (37), respectively. The critic NN weight tuning laws are updated by (46). If $|P_{I}|\in PE$ and $|P_{c}|\in PE$ , then the closed-loop system, system identifier estimation error ${\tilde{W}}_{I}$ , and critic estimation error ${\tilde{W}}_{c}$ are uniformly ultimately bounded (UUB). Moreover, the approximated $H_{\infty }$ control pair given by (36) and (37) are close to the optimal control pair $u^{★}$ and $d^{★}$ within a small region $b_{u}$ and $b_{d}$ , that is, $∥\hat{u}-u^{★}∥\leq b_{u}$ and $∥\hat{d}-d^{★}∥\leq b_{d}$ , where $b_{u}$ and $b_{d}$ are positive constants.*

**Proof.**  We consider the Lyapunov function as follows:

(56) $$\begin{array}{cc} J(t) & =\frac{1}{2}tr\left\{{\tilde{W}}_{I}^{T}\left(t\right)\gamma_{1}^{-1}{\tilde{W}}_{I}\left(t\right)\right\}+\frac{1}{2}{\tilde{W}}_{c}^{T}\left(t\right)\gamma_{2}^{-1}{\tilde{W}}_{c}\left(t\right)+\gamma_{3}x^{T}x+\gamma_{4}V^{★}\left(x\right)\\ \; & \;\;\;\;\;\;+\gamma_{5}tr\left\{V_{I}^{T}V_{I}\right\}+\gamma_{6}V_{c}^{T}V_{c}\\ \; & =J_{1}+J_{2}+J_{3}+J_{4}+J_{5}+J_{6}, \end{array}$$

where $\gamma_{3}$ , $\gamma_{4}$ , $\gamma_{5}$ and $\gamma_{6}$ are positive constants.

By applying matrix operations, we can obtain the following:

(57) $$\begin{array}{cc} \; & {\dot{\tilde{W}}}_{I}=-\gamma_{1}|P_{I}\left|[\right|P_{I}\left|{\tilde{W}}_{I}-V_{I}\right],\\ \; & {\dot{\tilde{W}}}_{c}=-\gamma_{2}|P_{c}\left|[\right|P_{c}\left|{\tilde{W}}_{c}-V_{c}\right]. \end{array}$$

According to Definition 1, $|P_{I}|\;\in PE$ and $|P_{c}|\in PE$ imply that $\int_{t}^{t+T}|P_{I}{|}^{2}dr\geq \delta_{I}>0$ and $\int_{t}^{t+T}|P_{c}{|}^{2}dr\geq \delta_{c}>0$ . Substituting (19), (43), and using Young’s inequality $ab\leq a^{2}\eta /2+b^{2}/2\eta$ with η>0 , we have

(58) $$\begin{array}{cc} {\dot{J}}_{1}\; & =tr\left\{-{\tilde{W}}_{I}^{T}|P_{I}{|}^{2}{\tilde{W}}_{I}+\left|P_{I}\right|{\tilde{W}}_{I}^{T}V_{I}\right\}\leq -\left(\delta_{I}-\frac{1}{2\eta }\right){∥{\tilde{W}}_{I}∥}^{2}+\frac{\eta }{2}b_{P_{I}^{*}}^{2}{∥|P_{I}|v_{I}∥}^{2}, \end{array}$$

(59) $$\begin{array}{cc} {\dot{J}}_{2}\; & =-{\tilde{W}}_{c}^{T}|P_{c}{|}^{2}{\tilde{W}}_{c}+\left|P_{c}\right|{\tilde{W}}_{c}^{T}V_{c}\leq -\left(\delta_{c}-\frac{1}{2\eta }\right){∥{\tilde{W}}_{c}∥}^{2}+\frac{\eta }{2}b_{P_{c}^{*}}^{2}{∥|P_{c}|v_{c}∥}^{2}. \end{array}$$

where $∥P_{I}^{*}∥\leq b_{P_{I}^{*}}$ , $∥P_{c}^{*}∥\leq b_{P_{c}^{*}}$ .

For $J_{3}$ and $J_{4}$ ,

(60) $$\begin{array}{cc} {\dot{J}}_{3}+{\dot{J}}_{4} & =2\gamma_{3}x^{T}\dot{x}+\gamma_{4}{\dot{V}}^{★}\left(x\right)\\ & =2\gamma_{3}x^{T}\left[f\left(x\right)+g\left(x\right)\hat{u}+G\left(x\right)\hat{d}-g\left(x\right)u^{★}+g\left(x\right)u^{★}\;-G\left(x\right)d^{★}+G\left(x\right)d^{★}\right]\\ & \;\;\;\;+\gamma_{4}\left(-x^{T}Qx-u^{★T}Ru^{★}+\kappa^{2}d^{★T}d^{★}\right)\\ \; & =2\gamma_{3}x^{T}[f\left(x\right)+g\left(x\right)(-\frac{1}{2}R^{-1}{\hat{g}}^{T}\left(x\right)\nabla \theta_{c}^{T}\left(x\right){\hat{W}}_{c}+\frac{1}{2}R^{-1}g^{T}\left(x\right)(\nabla \theta_{c}^{T}\left(x\right)W_{c}\\ \; & \;\;\;\;+\nabla \epsilon_{v}))+g\left(x\right)u^{★}+G\left(x\right)(\frac{1}{2\kappa^{2}}{\hat{G}}^{T}\left(x\right)\nabla \theta_{c}^{T}\left(x\right){\hat{W}}_{c}-\frac{1}{2\kappa^{2}}G^{T}\left(x\right)(\nabla \theta_{c}^{T}\left(x\right)W_{c}\\ & \;\;\;\;+\nabla \epsilon_{v}))+G\left(x\right)d^{★}]+\gamma_{4}\left(-x^{T}Qx-u^{★T}Ru^{★}+\kappa^{2}d^{★T}d^{★}\right). \end{array}$$

Since $g^{T}\nabla \theta_{c}^{T}W_{c}-{\hat{g}}^{T}\nabla \theta_{c}^{T}{\hat{W}}_{c}=g^{T}\nabla \theta_{c}^{T}{\tilde{W}}_{c}+{\tilde{g}}^{T}\nabla \theta_{c}^{T}{\hat{W}}_{c}$ , and $-G^{T}\nabla \theta_{c}^{T}W_{c}+{\hat{G}}^{T}\nabla \theta_{c}^{T}{\hat{W}}_{c}=$ $-G^{T}\nabla \theta_{c}^{T}{\tilde{W}}_{c}-{\tilde{G}}^{T}\nabla \theta_{c}^{T}{\hat{W}}_{c}$ , (60) can be rewritten as

(61) $$\begin{array}{cc} {\dot{J}}_{3}+{\dot{J}}_{4}= & 2\gamma_{3}x^{T}[f\left(x\right)+g\left(x\right)(\frac{1}{2}R^{-1}g^{T}\left(x\right)\nabla \theta_{c}^{T}\left(x\right){\tilde{W}}_{c}+\frac{1}{2}R^{-1}{\tilde{g}}^{T}\left(x\right)\nabla \theta_{c}^{T}\left(x\right){\hat{W}}_{c}\\ & +\frac{1}{2}R^{-1}g^{T}\left(x\right)\nabla \varepsilon_{v})+g\left(x\right)u^{★}+G\left(x\right)(-\frac{1}{2\kappa^{2}}G^{T}\left(x\right)\nabla \theta_{c}^{T}\left(x\right){\tilde{W}}_{c}\\ & -\frac{1}{2\kappa^{2}}{\tilde{G}}^{T}\left(x\right)\nabla \theta_{c}^{T}\left(x\right){\hat{W}}_{c}-\frac{1}{2\kappa^{2}}G^{T}\left(x\right)\nabla \epsilon_{v})+G\left(x\right)d^{★}]\\ & +\gamma_{4}\left(-x^{T}Qx-u^{★T}Ru^{★}+\kappa^{2}d^{★T}d^{★}\right)\\ \leq & -\left[\gamma_{4}\lambda_{m}\left(Q\right)-2\gamma_{3}b_{f}-4\eta \right]{∥x∥}^{2}+[\frac{1}{2\eta }\gamma_{3}^{2}b_{g}^{2}b_{\omega }^{2}b_{\nabla \theta_{c}}^{2}\lambda_{M}^{2}\left(R^{-1}\right)\\ & +\frac{1}{2\eta \kappa^{4}}\gamma_{3}^{2}b_{G}^{2}b_{\omega }^{2}b_{\nabla \theta_{c}}^{2}]{∥{\tilde{W}}_{I}∥}^{2}+\left[\frac{1}{2\eta }\gamma_{3}^{2}b_{g}^{4}b_{\nabla \theta_{c}}^{2}\lambda_{M}^{2}\left(R^{-1}\right)+\frac{1}{2\eta \kappa^{4}}\gamma_{3}^{2}b_{G}^{4}b_{\nabla \theta_{c}}^{2}\right]{∥{\tilde{W}}_{c}∥}^{2}\\ & -\left[\gamma_{4}\lambda_{m}\left(R\right)-2\gamma_{3}^{2}b_{g}^{2}/\eta \right]{∥u^{★}∥}^{2}+\left[\frac{1}{2\eta }\gamma_{3}^{2}b_{g}^{4}\lambda_{M}^{2}\left(R^{-1}\right)+\frac{\gamma_{3}^{2}}{\kappa^{4}}b_{G}^{4}\right]{∥\nabla \epsilon_{v}∥}^{2}\\ & +\left[\gamma_{4}\kappa^{2}+2\gamma_{3}^{2}b_{G}^{2}/\eta \right]{∥d^{★}∥}^{2}, \end{array}$$

where $b_{\omega }=∥{\hat{W}}_{c}∥$ is a bounded variable.

Recall that $V_{I}=P_{I}^{*}v_{I}$ and ${\dot{v}}_{I}=-l_{I}v_{I}+\theta_{If}\epsilon_{Tf}^{T}$ , thus

(62) $$\begin{array}{cc} {\dot{J}}_{5}\leq & 2\gamma_{5}b_{P_{I}^{*}}^{2}∥v_{I}^{T}{\dot{v}}_{I}∥=2\gamma_{5}b_{P_{I}^{*}}^{2}∥v_{I}^{T}\left[-l_{I}v_{I}+\theta_{If}\epsilon_{Tf}^{T}\right]∥\\ \leq & -\left(2\gamma_{5}b_{P_{I}^{*}}^{2}l_{I}-b_{P_{I}^{*}}^{2}\eta \right){∥v_{I}∥}^{2}+\frac{1}{\eta }\gamma_{5}^{2}b_{P_{I}^{*}}^{2}{∥\theta_{If}\epsilon_{Tf}^{T}∥}^{2}. \end{array}$$

Since ${\dot{v}}_{c}=-l_{c}v_{c}+\Theta \epsilon_{HJI}^{T}$ . Hence, the last term of (56) can be given as

(63) $$\begin{array}{cc} {\dot{J}}_{6}\leq & 2\gamma_{6}b_{P_{c}^{*}}^{2}∥v_{c}^{T}{\dot{v}}_{c}∥\\ = & 2\gamma_{6}b_{P_{c}^{*}}^{2}∥v_{c}^{T}\left[-l_{c}v_{c}+\Theta \epsilon_{HJI}^{T}\right]∥\\ \leq & -\left(2\gamma_{6}l_{c}b_{P_{c}^{*}}^{2}-5b_{P_{c}^{*}}^{2}\eta \right){∥v_{c}∥}^{2}+\frac{1}{\eta }\gamma_{6}^{2}b_{P_{c}^{*}}^{2}b_{W_{c}}^{2}b_{\nabla \theta_{c}}^{2}{∥\Theta ∥}^{2}{∥\epsilon_{T}∥}^{2}\\ & +\frac{1}{\eta }\gamma_{6}^{2}b_{P_{c}^{*}}^{2}b_{\nabla \theta_{c}}^{2}b_{W_{c}}^{2}{∥\theta_{I}∥}^{2}{∥\Theta ∥}^{2}{∥{\tilde{W}}_{I}∥}^{2}+\frac{1}{\eta }\gamma_{6}^{2}b_{P_{c}^{*}}^{2}b_{f}^{2}b_{\nabla \epsilon_{v}}^{2}{∥\Theta ∥}^{2}{∥x∥}^{2}\\ & +\frac{1}{4\eta }\gamma_{6}^{2}b_{P_{c}^{*}}^{2}b_{g}^{2}b_{\varpi 1}^{2}b_{\nabla \theta_{c}}^{2}b_{\omega }^{2}\lambda_{M}^{2}\left(R^{-1}\right){∥\Theta ∥}^{2}{∥\nabla \epsilon_{v}∥}^{2}\\ & +\frac{1}{4\eta \kappa^{4}}\gamma_{6}^{2}b_{P_{c}^{*}}^{2}b_{G}^{2}b_{\varpi 2}^{2}b_{\nabla \theta_{c}}^{2}b_{\omega }^{2}{∥\Theta ∥}^{2}{∥\nabla \epsilon_{v}∥}^{2}. \end{array}$$

where $b_{\varpi 1}=∥{\hat{W}}_{g}\theta_{g}∥$ and $b_{\varpi 2}=∥{\hat{W}}_{G}\theta_{G}∥$ are bounded variables. Consequently, we substitute (58), (59), and (61)–(63) into (56); thus, we have

(64) $$\begin{array}{cc} \dot{J}\left(t\right) & ={\dot{J}}_{1}+{\dot{J}}_{2}+{\dot{J}}_{3}+{\dot{J}}_{4}+{\dot{J}}_{5}+{\dot{J}}_{6}\\ \; & \leq -(\delta_{I}-\frac{1}{2\eta }-\frac{1}{2\eta }\gamma_{3}^{2}b_{g}^{2}b_{\omega }^{2}b_{\nabla \theta_{c}}^{2}\lambda_{M}^{2}\left(R^{-1}\right)-\frac{1}{2\eta \kappa^{4}}\gamma_{3}^{2}b_{G}^{2}b_{\omega }^{2}b_{\nabla \theta_{c}}^{2}\\ \; & \;\;\;-\frac{1}{\eta }\gamma_{6}^{2}b_{P_{c}^{*}}^{2}b_{\nabla \theta_{c}}^{2}b_{W_{c}}^{2}{∥\theta_{I}∥}^{2}{∥\Theta ∥}^{2}){∥{\tilde{W}}_{I}∥}^{2}-\left(\gamma_{4}\lambda_{m}\left(R\right)\;-\;\frac{2}{\eta }\gamma_{3}^{2}b_{g}^{2}\right){∥u^{★}∥}^{2}\\ \; & \;\;\;-\left(\delta_{c}-\frac{1}{2\eta }-\frac{1}{2\eta }\gamma_{3}^{2}b_{g}^{4}b_{\nabla \theta_{c}}^{2}\lambda_{M}^{2}\left(R^{-1}\right)-\frac{1}{2\eta \kappa^{4}}\gamma_{3}^{2}b_{G}^{4}b_{\nabla \theta_{c}}^{2}\right){∥{\tilde{W}}_{c}∥}^{2}\\ \; & \;\;\;-\left(\gamma_{4}\lambda_{m}\left(Q\right)-2\gamma_{3}b_{f}-4\eta -\frac{1}{\eta }\gamma_{6}^{2}b_{P_{c}^{*}}^{2}b_{f}^{2}b_{\nabla \epsilon_{v}}^{2}{∥\Theta ∥}^{2}\right){∥x∥}^{2}+\frac{1}{\eta }\gamma_{5}^{2}b_{P_{I}^{*}}^{2}{∥\phi_{If}\epsilon_{Tf}^{T}∥}^{2}\\ & \;\;\;-\left(2\gamma_{5}b_{P_{I}^{*}}^{2}l_{I}-b_{P_{I}^{*}}^{2}\eta -\frac{\eta }{2}b_{P_{I}^{*}}^{2}{\left|P_{I}\right|}^{2}\right){∥v_{I}∥}^{2}-\left(2\gamma_{6}l_{c}b_{P_{c}^{*}}^{2}-5b_{P_{c}^{*}}^{2}\eta -\frac{\eta }{2}b_{P_{I}^{*}}^{2}{\left|P_{I}\right|}^{2}\right){∥v_{c}∥}^{2}\\ & \;\;\;+(\frac{1}{2\eta }\gamma_{3}^{2}b_{g}^{4}\lambda_{M}^{2}\left(R^{-1}\right)+\frac{\gamma_{3}^{2}}{\kappa^{4}}b_{G}^{4}+\frac{1}{4\eta }\gamma_{6}^{2}b_{P_{c}^{*}}^{2}b_{g}^{2}b_{\varpi 1}^{2}b_{\nabla \theta_{c}}^{2}b_{\omega }^{2}\lambda_{M}^{2}\left(R^{-1}\right){∥\Theta ∥}^{2}\\ & \;\;\;+\frac{1}{4\eta \kappa^{4}}\gamma_{6}^{2}b_{P_{c}^{*}}^{2}b_{G}^{2}b_{\varpi 2}^{2}b_{\nabla \theta_{c}}^{2}b_{\omega }^{2}{∥\Theta ∥}^{2}){∥\nabla \epsilon_{v}∥}^{2}+\frac{1}{\eta }\gamma_{6}^{2}b_{P_{c}^{*}}^{2}b_{W_{c}}^{2}b_{\nabla \theta_{c}}^{2}{∥\Theta ∥}^{2}{∥\epsilon_{T}∥}^{2}. \end{array}$$

We choose the parameters $\gamma_{3}$ , $\gamma_{4}$ , $\gamma_{5}$ , $\gamma_{6}$ and η , fulfilling the following conditions

$$\begin{array}{cc} \; & \eta >\max\{(\kappa^{4}+\kappa^{4}\gamma_{3}^{2}b_{g}^{2}b_{\omega }^{2}b_{\nabla \theta_{c}}^{2}\lambda_{M}^{2}\left(R^{-1}\right)+\gamma_{3}^{2}b_{G}^{2}b_{\omega }^{2}b_{\nabla \theta_{c}}^{2}+2\kappa^{4}\gamma_{6}^{2}b_{P_{c}^{*}}^{2}b_{\nabla \theta_{c}}^{2}b_{W_{c}}^{2}\\ & \;\;\;\;\;\;\;\;\;\;\;\;\;\;\;\;{∥\theta_{I}∥}^{2}{∥\Theta ∥}^{2})/2\kappa^{4}\delta_{I},\left(\kappa^{4}+\kappa^{4}\gamma_{3}^{2}b_{g}^{4}b_{\nabla \theta_{c}}^{2}\lambda_{M}^{2}\left(R^{-1}\right)+\kappa^{4}\gamma_{3}^{2}b_{G}^{4}b_{\nabla \theta_{c}}^{2}\right)/2\kappa^{4}\delta_{c}\},\\ \; & \gamma_{3}<\sqrt{\gamma_{4}\eta \lambda_{m}\left(R\right)/2b_{g}^{2}},\\ \; & \gamma_{4}>\left\{2\gamma_{3}b_{f}-4\eta -\frac{1}{\eta }\gamma_{6}^{2}b_{P_{c}^{*}}^{2}b_{f}^{2}b_{\nabla \epsilon_{v}}^{2}{∥\Theta ∥}^{2}\right\}/\lambda_{m}\left(Q\right),\\ \; & \gamma_{5}>(\eta +\frac{\eta }{2}|P_{I}{|}^{2})/2l_{I},\\ \; & \gamma_{6}>(5\eta -\frac{\eta }{2}|P_{c}{|}^{2})/2l_{c}. \end{array}$$

Then, (64) can be further presented as

(65) $$\begin{array}{cc} \dot{J}\left(t\right) & \leq -k_{1}{∥{\tilde{W}}_{I}∥}^{2}-k_{2}{∥{\tilde{W}}_{c}∥}^{2}-k_{3}{∥x∥}^{2}-k_{4}{∥v_{I}∥}^{2}-k_{5}{∥v_{c}∥}^{2}+b_{\gamma }, \end{array}$$

where $k_{1}$ , $k_{2}$ , $k_{3}$ , $k_{4}$ , $k_{5}$ and $b_{\gamma }$ are positive constants

$$\begin{array}{cc} k_{1}= & \delta_{I}-\frac{1}{2\eta }-\frac{1}{2\eta }\gamma_{3}^{2}b_{g}^{2}b_{\omega }^{2}b_{\nabla \theta_{c}}^{2}\lambda_{M}^{2}\left(R^{-1}\right)-\frac{1}{2\eta \kappa^{4}}\gamma_{3}^{2}b_{G}^{2}b_{\omega }^{2}b_{\nabla \theta_{c}}^{2}-\frac{1}{\eta }\gamma_{6}^{2}b_{P_{c}^{*}}^{2}b_{\nabla \theta_{c}}^{2}b_{W_{c}}^{2}{∥\theta_{I}∥}^{2}{∥\Theta ∥}^{2},\\ k_{2}= & \delta_{c}-\frac{1}{2\eta }-\frac{1}{2\eta }\gamma_{3}^{2}b_{g}^{4}b_{\nabla \theta_{c}}^{2}\lambda_{M}^{2}\left(R^{-1}\right)-\frac{1}{2\eta \kappa^{4}}\gamma_{3}^{2}b_{G}^{4}b_{\nabla \theta_{c}}^{2},\\ k_{3}= & \gamma_{4}\lambda_{m}\left(Q\right)-2\gamma_{3}b_{f}-4\eta -\frac{1}{\eta }\gamma_{6}^{2}b_{P_{c}^{*}}^{2}b_{f}^{2}b_{\nabla \epsilon_{v}}^{2}{∥\Theta ∥}^{2},\\ k_{4}= & 2\gamma_{5}b_{P_{I}^{*}}^{2}l_{I}-b_{P_{I}^{*}}^{2}\eta -\frac{\eta }{2}b_{P_{I}^{*}}^{2}|P_{I}{|}^{2},\;\;k_{5}=2\gamma_{6}l_{c}b_{P_{c}^{*}}^{2}-5b_{P_{c}^{*}}^{2}\eta -\frac{\eta }{2}b_{P_{c}^{*}}^{2}{\left|P_{c}\right|}^{2},\\ b_{\gamma }= & (\frac{1}{2\eta }\gamma_{3}^{2}b_{g}^{4}\lambda_{M}^{2}\left(R^{-1}\right)+\frac{\gamma_{3}^{2}}{\kappa^{4}}b_{G}^{4}+\frac{1}{4\eta }\gamma_{6}^{2}b_{P_{c}^{*}}^{2}b_{g}^{2}b_{\varpi 1}^{2}b_{\nabla \theta_{c}}^{2}b_{\omega }^{2}\lambda_{M}^{2}\left(R^{-1}\right){∥\Theta ∥}^{2}+\frac{1}{\eta }\gamma_{5}^{2}b_{P_{I}^{*}}^{2}{∥\phi_{If}\epsilon_{Tf}^{T}∥}^{2}\\ + & \frac{1}{4\eta \kappa^{4}}\gamma_{6}^{2}b_{P_{c}^{*}}^{2}b_{G}^{2}b_{\varpi 2}^{2}b_{\nabla \theta_{c}}^{2}b_{\omega }^{2}{∥\Theta ∥}^{2}){∥\nabla \epsilon_{v}∥}^{2}+\frac{1}{\eta }\gamma_{6}^{2}b_{P_{c}^{*}}^{2}b_{W_{c}}^{2}b_{\nabla \theta_{c}}^{2}{∥\Theta ∥}^{2}{∥\epsilon_{T}∥}^{2}. \end{array}$$

Thus, $\dot{J}\left(t\right)$ is negative if

$$\begin{array}{c} ∥{\tilde{W}}_{I}∥>\sqrt{b_{\gamma }/k_{1}},∥{\tilde{W}}_{c}∥>\sqrt{b_{\gamma }/k_{2}},∥x∥>\sqrt{b_{\gamma }/k_{3}},∥\Xi_{I}∥>\sqrt{b_{\gamma }/k_{4}},∥\Xi_{c}∥>\sqrt{b_{\gamma }/k_{5}}, \end{array}$$

which implies that the NN weight estimation errors ${\tilde{W}}_{I}$ , ${\tilde{W}}_{c}$ and the system state *x* are all UUB.

Lastly, the error between the proposed $H_{\infty }$ control pair and the ideal one are written as

$$\begin{array}{cc} \hat{u}-u^{★}= & -\frac{1}{2}R^{-1}{\left[{\hat{W}}_{g}\theta_{g}\left(x\right)\right]}^{T}\nabla \theta_{c}^{T}\left(x\right){\hat{W}}_{c}+\frac{1}{2}R^{-1}g^{T}\left(\nabla \theta_{c}^{T}\left(x\right)W_{c}+\nabla \epsilon_{v}\right)\\ = & \frac{1}{2}R^{-1}g^{T}\nabla \theta_{c}^{T}\left(x\right){\tilde{W}}_{c}+\frac{1}{2}R^{-1}{\left[g-{\hat{W}}_{g}\theta_{g}\left(x\right)\right]}^{T}\nabla \theta_{c}^{T}\left(x\right)W_{c}\\ \; & -\frac{1}{2}R^{-1}{\left[g-{\hat{W}}_{g}\theta_{g}\left(x\right)\right]}^{T}\nabla \theta_{c}^{T}\left(x\right){\tilde{W}}_{c}+\frac{1}{2}R^{-1}g^{T}\nabla \epsilon_{v}, \end{array}$$

$$\begin{array}{cc} \hat{d}-d^{★}= & \frac{1}{2\kappa^{2}}{\left[{\hat{W}}_{G}\theta_{G}\left(x\right)\right]}^{T}\nabla \theta_{c}^{T}\left(x\right){\hat{W}}_{c}-\frac{1}{2\kappa^{2}}G^{T}\left(\nabla \theta_{c}^{T}\left(x\right)W_{c}+\nabla \epsilon_{v}\right)\\ = & -\frac{1}{2\kappa^{2}}G^{T}\nabla \theta_{c}^{T}\left(x\right){\tilde{W}}_{c}+\frac{1}{2\kappa^{2}}{\left[G-{\hat{W}}_{G}\theta_{G}\left(x\right)\right]}^{T}\nabla \theta_{c}^{T}\left(x\right)W_{c}\\ \; & +\frac{1}{2\kappa^{2}}{\left[G-{\hat{W}}_{G}\theta_{G}\left(x\right)\right]}^{T}\nabla \theta_{c}^{T}\left(x\right){\tilde{W}}_{c}-\frac{1}{2\kappa^{2}}{\left[{\hat{W}}_{G}\theta_{G}\left(x\right)\right]}^{T}\nabla \epsilon_{v}, \end{array}$$

which further implies the following fact

$$\begin{array}{cc} \lim_{t\to +\infty }∥\hat{u}\;-\;u^{★}∥\leq & \frac{1}{2}\lambda_{M}\left(R^{-1}\right)\{b_{g}\left(b_{\nabla \theta_{c}}∥{\tilde{W}}_{c}∥\;+\;b_{\nabla \epsilon_{v}}\right)+b_{\nabla \theta_{c}}b_{W_{c}}\left(∥{\tilde{W}}_{I}∥+∥b_{g}∥\right)\\ \; & +b_{\nabla \theta_{c}}∥{\tilde{W}}_{c}∥\left(∥{\tilde{W}}_{I}∥+∥b_{g}∥\right)\}\leq b_{u}, \end{array}$$

$$\begin{array}{cc} \lim_{t\to +\infty }∥\hat{d}\;-\;d^{★}∥\leq & \frac{1}{2\kappa^{2}}\{b_{G}\left(b_{\nabla \theta_{c}}∥{\tilde{W}}_{c}∥\;+\;b_{\nabla \epsilon_{v}}\right)+b_{\nabla \theta_{c}}b_{W_{c}}\left(∥{\tilde{W}}_{I}∥+∥b_{G}∥\right)\\ \; & +b_{\nabla \theta_{c}}∥{\tilde{W}}_{c}∥\left(∥{\tilde{W}}_{I}∥+∥b_{G}∥\right)\}\leq b_{d}, \end{array}$$

where $b_{u}>0$ and $b_{d}>0$ are constants determined by the identifier NN estimation error ${\tilde{W}}_{I}$ and the critic NN estimation error ${\tilde{W}}_{c}$ . It proves that the approximate $H_{\infty }$ control pair can converge to a set around the optimal solution.

This completes the proof. □

## 6. Numerical Simulation

This section aims to verify the effectiveness of the proposed KREM-based IC learning approach for optimal robust control. We consider the following NCT system [12]

(66) $$\begin{array}{c} \dot{x}=f\left(x\right)+g\left(x\right)u+G\left(x\right)d, \end{array}$$

where $f\left(x\right)=\left[\begin{array}{c} \;\;\;\;\;\;\;\;\;\;\;\;\;\;\;\;-x_{1}+x_{2}\\ -0.5x_{1}-0.5x_{2}\left(1-{\left(\cos\left(2x_{1}\right)+2\right)}^{2}\right) \end{array}\right]$ , $g\left(x\right)=\left[\begin{array}{c} \;\;\;\;\;\;\;0\\ \cos(2x_{1})+2 \end{array}\right]$ , $G\left(x\right)=\left[\begin{array}{c} \;\;\;\;\;\;\;0\\ \sin(4x_{1})+2 \end{array}\right]$ .

We choose the regressor of identifier NN as

$$\theta_{I}\left(x,u\right)={\left[x_{1},x_{2},x_{2}\left(1-{\left(\cos\left(2x_{1}\right)+2\right)}^{2}\right),u\cos\left(2x_{1}\right),u,d\sin\left(4x_{1}\right),d\right]}^{T},$$

with the unknown identifier weight matrix given by

$$W_{I}=\left[\begin{array}{ccccccc} -1 & 1 & 0 & 0 & 0 & 0 & 0\\ -0.5\; & 0 & -0.5\; & 1 & 2 & 1 & 2 \end{array}\right].$$

The activation function in (33) for the critic NN is selected as

$$\theta_{c}\left(x,u\right)={\left[x_{1}^{2},x_{1}x_{2},x_{2}^{2}\right]}^{T}.$$

The ideal critic NN weights were $W_{c}={\left[0.5,0,1\right]}^{T}$ .

In this numerical example, several other parameters are set as follows: the initial values of the system states are $x_{1}\left(0\right)=3$ and $x_{2}\left(0\right)=-1$ . $Q=I_{2}$ and R=1 . The filter coefficients are ρ=0.001 , $l_{I}=0.1$ , $l_{c}=20$ , $\gamma_{1}=800$ , $\gamma_{2}=200diag\left\{0.3,1,1\right\}$ . It is important to note that in this simulation, there is no need to add noise to the control input u(t) to ensure the PE condition. This condition is often necessary for many existing ADP-based control methods to ensure that $\theta_{I}\left(t\right)\in PE$ and Θ(t)∈PE .

**Figure 2:**
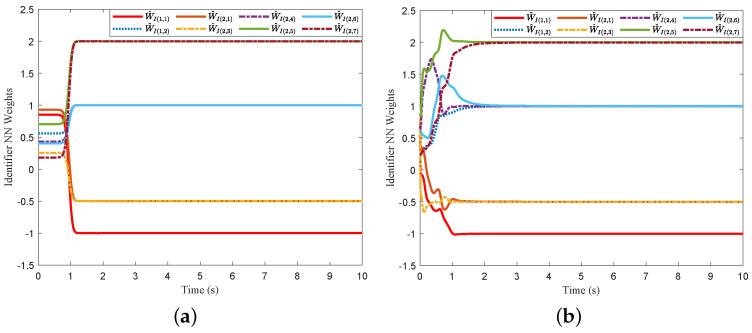
Comparison of the convergence of identifier NN’s weights ${\hat{W}}_{I}$ : (**a**) KREM-based method; (**b**) KRE-based method in [12].

**Figure 3:**
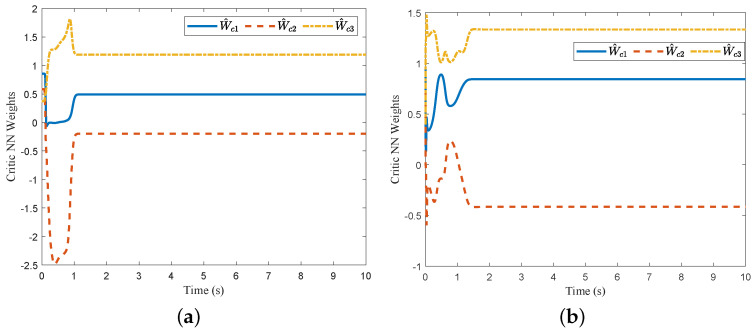
Comparison of the convergence of critic NN’s weights ${\hat{W}}_{c}$ : (**a**) KREM-based method; (**b**) KRE-based method in [12].

**Figure 4:**
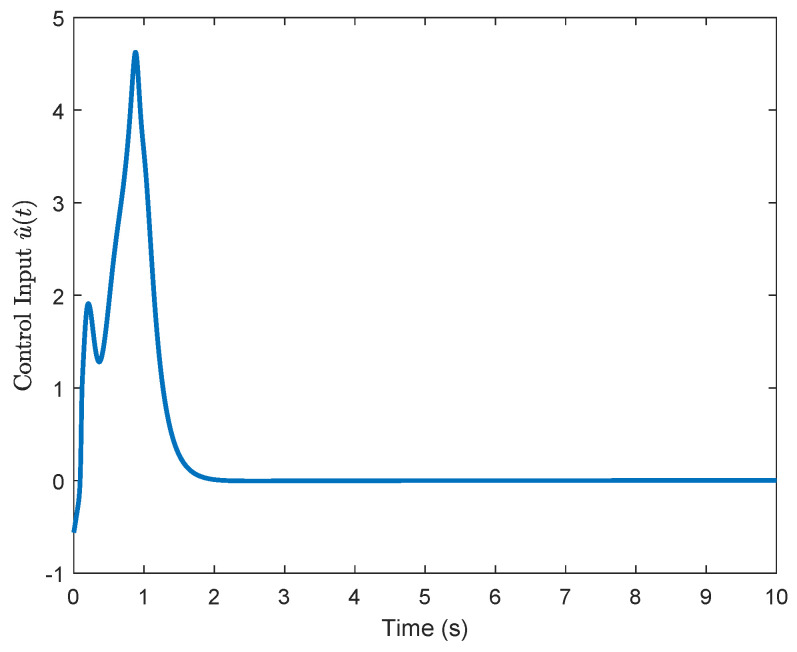
Evolution of the approximate control input $\hat{u}$ .

**Figure 5:**
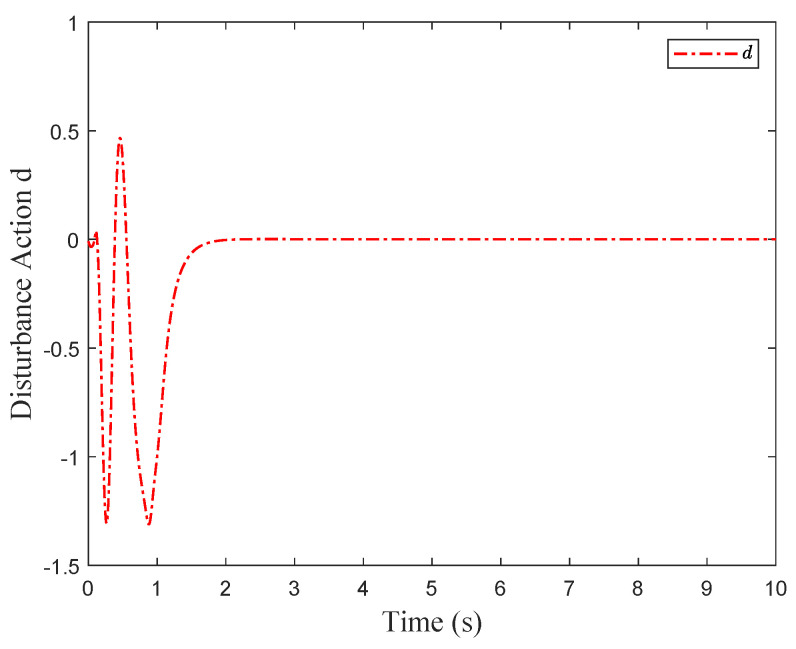
Disturbance action *d*.

**Figure 6:**
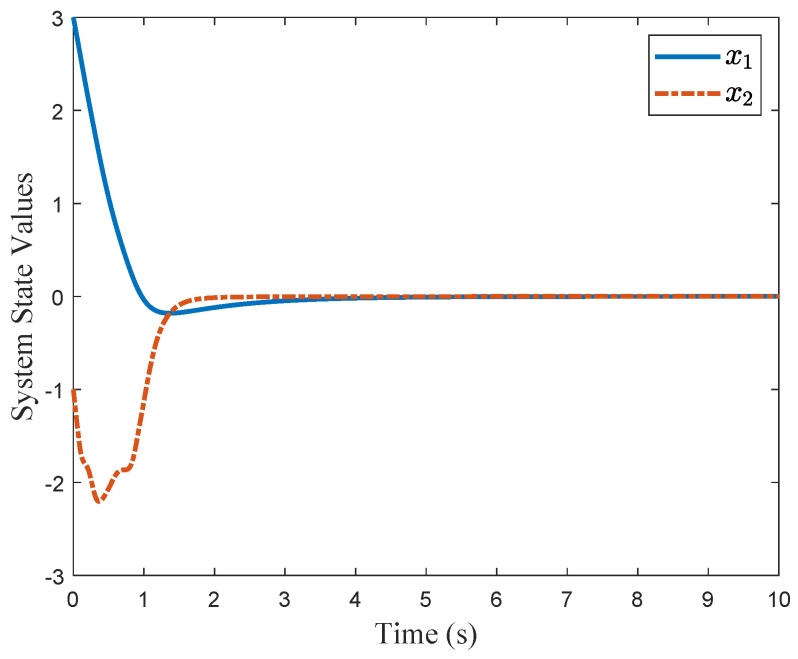
Trajectories of the system states $x={\left[x_{1},x_{2}\right]}^{T}$ .

For comparison, we consider the Kreisselmeier’s Regressor Extension (KRE) based identifier-critic network framework [12] for the system (66). Figure 2 and Figure 3 display the convergence of the identifier NN weights and the critic NN weights, respectively, under our KREM-based optimal robust control method and the KRE-based control method [12]. As illustrated in Figure 2, the KREM-based ADP method proposed in this paper exhibits faster convergence compared to the KRE-based ADP method. Furthermore, it demonstrates element-wise monotonicity, thus preventing oscillations and peaking in the learning curve. The trajectories of the approximate control input $\hat{u}$ and the estimated disturbance $\hat{d}$ are presented in Figure 4 and Figure 5, respectively. By applying the optimal $H_{\infty }$ control pair, the system states are stabilized, as depicted in Figure 6.

## 7. Conclusions

This paper presents a novel adaptive learning approach using neural networks (NNs) to address the problem of optimal robust control for nonlinear continuous-time systems with unknown dynamics. The approach involves employing a system identifier that utilizes NNs and parameter estimation techniques to approximate the unknown system matrices and disturbances. Additionally, a critic NNs learning structure is introduced to obtain an approximate controller that corresponds to the optimal control problem. Unlike existing identifier-critic NNs learning control methods, this approach incorporates adaptive tuning laws based on a regressor extension and mixing technique. These laws facilitate the learning of unknown parameters in the two NNs under relaxed persistence of excitation conditions. The convergence conditions of the proposed approach have been theoretically demonstrated. Finally, the effectiveness of the proposed learning control approach is validated via a simulation study.

## Data Availability

The data presented in this study are available on request from the corresponding author.
